# Enhancing Facial Projection: Efficacy and Safety of a Novel Filler Combining Cross‐Linked Sodium Hyaluronate Gel With Poly‐L‐Lactic Acid‐b‐PEG Microspheres for T‐Zone Augmentation

**DOI:** 10.1111/jocd.70003

**Published:** 2025-03-20

**Authors:** Shiwei Wang, Yinxian Qiu, Mengjie Zhu, Muyan Zou, Wei Chen, Guanqun Qiao, Jialun Li, Jiaxu Wu, Yujia Diao, Wei Cai

**Affiliations:** ^1^ Medical Department Imeik Technology Development Co. Ltd Beijing China; ^2^ Department of Medical Cosmetology Chongqing Xingchen Plastic Surgery Hospital Chongqing China; ^3^ Department of Medical Cosmetology Ningbo Jia He Plastic Surgery Hospital Ningbo China; ^4^ Department of Plastic Surgery The Second Affiliated Hospital of Nanjing Medical University Nanjing China; ^5^ Plastic Surgery Pikeli Medical Aesthetics Wuhan China

**Keywords:** collagen stimulator, eyebrows, forehead, hyaluronic acid, nose, poly‐L‐lactic acid, three‐dimensionality, T‐zone

## Abstract

**Background:**

The nose, eyebrow, and forehead are critical elements of the T‐zone, vital for enhancing facial three‐dimensionality. Many Asians seek a more contoured and sculpted facial appearance by T‐zone augmentation. The novel poly‐L‐lactic acid microsphere and hyaluronic acid suspension (PLLA‐b‐PEG/HA) has proven to be a safe and effective dermal filler, making it an appealing choice for individuals aiming for T‐zone augmentation.

**Objective:**

To retrospectively assess the efficacy and safety of PLLA‐b‐PEG/HA injection into the T‐zone for aesthetic enhancement.

**Methods:**

Fifteen participants were included in this study. A comprehensive clinical evaluation was performed, including measurements of total facial convexity angle and radix height, the FACE‐Q scales for satisfaction with the nose, forehead, and eyebrows, the Global Aesthetic Improvement Scale (GAIS), and a 7‐item satisfaction scale. Tissue in the peri‐eyebrow area was examined 12 months posttreatment for comparison with normal tissues.

**Results:**

Significant increases in radix height and total facial convexity angle were observed at each follow‐up visit (*p* < 0.05). FACE‐Q scores also showed significant improvements from baseline in the eyebrows, forehead, and nose at each posttreatment visit (*p* < 0.001). The GAIS improvement rate remained high, with 80.00% reported by participants and 86.67% by blinded evaluators 12 months after treatment. The satisfaction rate was 73.3% at this time interval. Pathological examinations demonstrated newly formed collagen fibers and microvessels, with no abnormal pathological structure.

**Conclusion:**

The PLLA‐b‐PEG/HA demonstrates efficacy in enhancing the T‐zone (nose, eyebrow, and forehead) with a favorable safety profile.

## 1 Introduction

The “T‐zone,” encompassing the forehead, nose, medial cheeks, and chin, is the fundamental backbone of a three‐dimensional facial profile [1]. A face with a prominently defined middle forehead and nasal root area is generally regarded as attractive [2]. Asian facial structures are typically broader with a shorter vertical dimension, characterized by a flat or concave medial maxilla and limited projection of the brow, nose, and chin [3]. Consequently, augmentation of the anterior projection of the T‐zone is commonly pursued to enhance facial three‐dimensionality in the Asian population [3].

The nose is pivotal in forming the three‐dimensional structure of the whole face. Commonly, Asian noses are smaller and flatter, leading individuals to seek rhinoplasty for a higher nasal radix, more pronounced nasal tips, and narrower nostril bases [4]. Similarly, the eyebrow arch and forehead are also essential for constructing full facial three‐dimensionality and enhancing projection. A wide, square forehead is deemed attractive [5]. Previous studies have shown that augmenting the forehead can often improve fine lines and skin texture, potentially reducing the need for additional skin rejuvenation treatments [6]. The eyebrow arch supports and contours the upper face. In East Asian populations, common facial characteristics include a low brow area, underdeveloped orbital bones, eyeball protrusion, and upper eyelid skin laxity, which is often regarded as less [7]. A high and full brow arch can also impart a three‐dimensional appearance, significantly enhancing overall facial esthetics. Additionally, antiaging remains another key focus in facial esthetics. It is widely recognized that the facial bones, particularly in the midface area, undergo continuous changes because of the bone absorption throughout the aging process [8, 9]. Skeletal augmentation of the T‐zone can address related issues, such as upper eyelid skin laxity, resulting in the rejuvenation of these areas and a youthful appearance [10].

Several methods are available for augmentation purposes, including surgical methods (internal browpexy, glabellar myoplasty, coronal forehead and eyebrow lift, rhinoplasty, etc.) and nonsurgical methods [11]. However, many potential patients are reluctant to undergo surgery [3]. For these individuals, reshaping the nose, forehead, and eyebrow area with injectable fillers could represent a suitable, noninvasive alternative to surgical procedures [12]. Recently, the FDA has approved several injectable fillers for use in facial esthetics, including hyaluronic acid (HA), calcium hydroxyapatite (CaHA), poly‐L‐lactic acid (PLLA), and polymethylmethacrylate (PMMA) [13]. Among these, the HA fillers are more frequently and widely used for facial restructuring and addressing structural deficiency in the projection of nose in young Asians compared with the Western population, which may be related to the Asian facial features mentioned above [14, 15]. Additionally, in recent years, HA has also been used to volumize and sculpt the forehead of Asians, achieving a convex and youthful esthetic [16]. PLLA is a synthetic polymer that is considered biocompatible and biodegradable. The PLLA degrades naturally in the human body along with the same metabolic pathway as lactic acid. The uneven distribution of PLLA particles is considered to be one of the causes of PLLA‐related AE, such as granuloma and nodule [17, 18]. The PLLA‐b‐PEG/HA hybrid filler mitigates these risks associated with traditional PLLA products. It provides both short‐term physical support and long‐term effects through tissue regeneration.

This study aims to retrospectively investigate the efficacy and safety of the novel PLLA‐b‐PEG/HA filler for augmenting the T‐zone (nose, forehead, and eyebrows).

## 2 Material and Method

### 2.1 Material

The cross‐linked sodium hyaluronate gel with PLLA‐b‐PEG microsphere was developed by Imeik Technology Development Co. Ltd., Beijing, China, and has been approved by the National Medical Products Administration (NMPA). The components were as follows: PLLA‐b‐PEG microsphere at 180 mg/mL, hyaluronate at 17 mg/mL, and lidocaine hydrochloride at 3 mg/mL.

### 2.2 Study Design

This retrospective study included 15 participants who received PLLA‐b‐PEG/HA injections in the eyebrows, nose, and forehead for esthetic improvement of the T‐zone. The inclusion criteria for this study were as follows: adults aged 18–60 years seeking T‐zone augmentation (nose, eyebrow, and forehead) to improve facial three‐dimensionality. Exclusion criteria included a history of allergic reactions to PLLA, HA, or related components, the presence of active skin infections or inflammatory conditions in the T‐zone, bleeding disorders, or ongoing use of anticoagulant medications that could not be paused. Pregnant or breastfeeding women, individuals with a history of facial trauma or surgeries in the T‐zone area within the last 12 months, and those unable to attend scheduled follow‐up visits were also excluded from the study. This study was conducted in compliance with the Declaration of Helsinki, and all participants gave informed consent before receiving treatment.

### 2.3 Treatment

Prior to injection, the skin in the treatment area was disinfected with iodine, and the anesthetic cream was applied to the injection site to minimize discomfort. The injections were performed using a top–down method, from the forehead to the nose. Coded injection points were employed to minimize potential risks and complications. The specific injection techniques, points, and associated cautions are detailed in Table 1. Participants received the follow‐up visit at 1, 3, 6, and 12 months after treatment.

**TABLE 1:** Techniques, cautions, and purposes of injection procedures for forehead, eyebrows, and nose.

Facial area	Technique	Cautions	Purpose
Forehead	Injection depth targeted above the periosteum; spaced locations along the Forehead	Avoiding key blood vessels in the forehead region, particularly the supratrochlear artery and the supraorbital artery	Enhance forehead contouring and volume
Eyebrows	Injection depth targeted above the periosteum; specific points along the lateral brow area		Expand volume and lift the eyebrow
Nose	Injection depth targeted above the periosteum; spaced locations along the asal dorsum	Avoiding key anatomical structures, particularly the dorsal nasal artery	Reshape and lift the nasaldorsum

### 2.4 Efficacy Assessment

Photographs of participants were captured using a DSLR camera (EOS M6; Canon) prior to and at 1, 3, 6, and 12 months postinjection. To ensure consistency, identical positioning, angles, and camera settings were maintained across all sessions.

#### 2.4.1 Total Facial Convexity Angle and Radix Height

**FIGURE 1:**
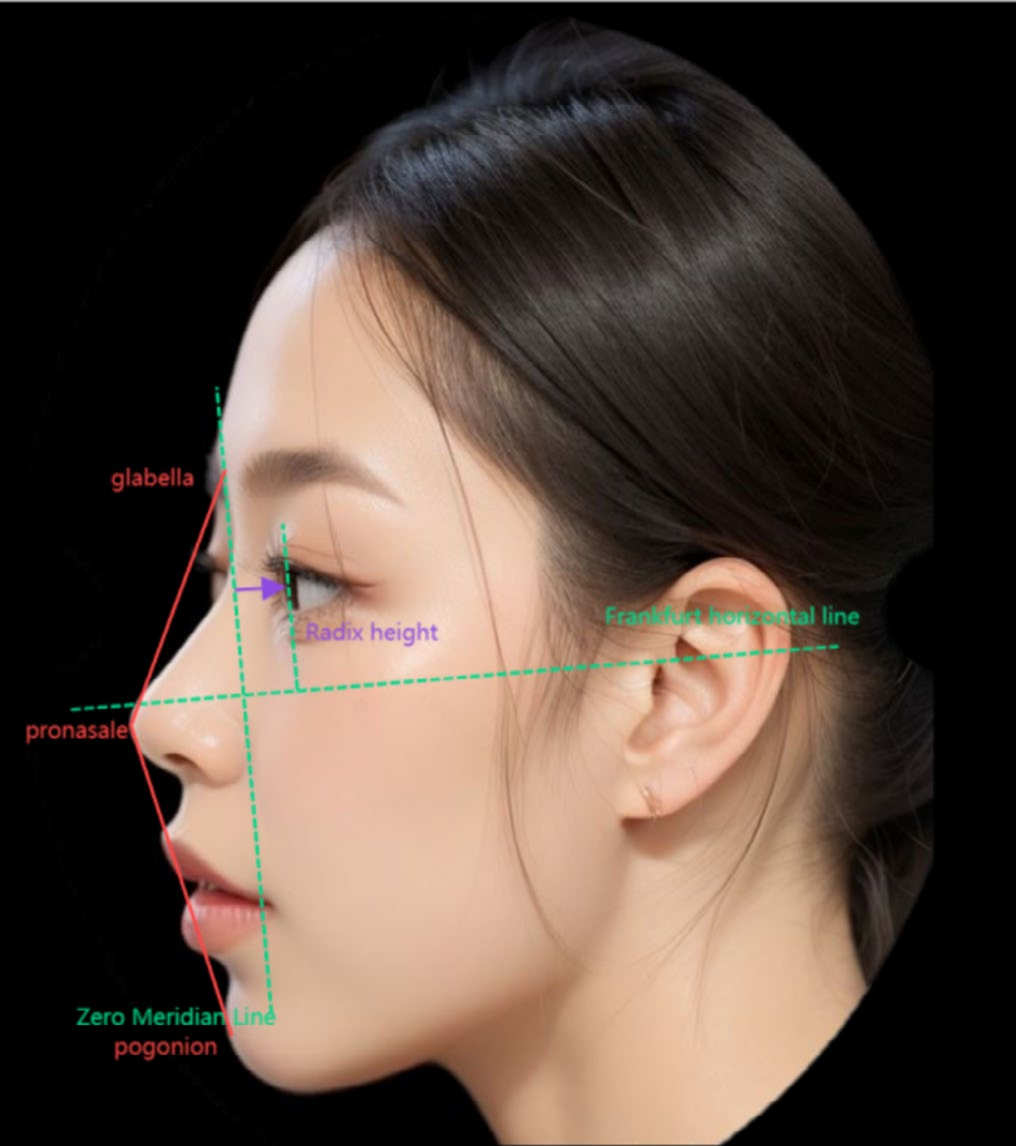
Schematic diagram of the total facial convexity angle (red) and radix height (purple). The Zero Meridian Line and Frankfurt Horizontal Line, shown in green, serve as references for facial profile analysis. These measurements are illustrated using an artificial intelligence‐generated model to enhance the visualization of facial proportions.

The total facial convexity angle (g‐prn‐pg) was defined by the configuration of three anatomical landmarks: the glabella (g), the pronasale (prn), and the pogonion (pg) (Figure 1). Radix height was determined by the linear distance from the median 0° line to the pupillary line, perpendicular to the Frankfort Horizontal (FH) plane (Figure 1). Measurements were taken before treatment and at 1, 3, 6, and 12 months posttreatment by an evaluating investigator (EI).

#### 2.4.2 Face‐Q Scale

The self‐reported FACE‐Q scales, assessing satisfaction with forehead, eyebrows, and nose, were administered pretreatment, and at 6 and 12 months posttreatment. Responses were scored on a Likert scale from 1 (“very dissatisfied”) to 4 (“very satisfied”). The scores were standardized to a Rasch‐transformed summary score ranging from 0 to 100, where higher scores indicate greater satisfaction.

#### 2.4.3 GAIS Improvement Rate

Esthetic improvement was assessed by both participants and the EI using the 5‐point Global Aesthetic Improvement Scale (GAIS) at 1, 3, 6, and 12 months posttreatment. Improvements were categorized from 3 (“improved”) to 5 (“very much improved”), with the improvement rate calculated as the percentage of participants achieving these scores.

#### 2.4.4 Satisfaction Rate

Participants assessed their satisfaction with the treatment outcomes using a 7‐item satisfaction scale at 1, 3, 6, and 12 months posttreatment. The satisfaction rate was defined as the proportion of participants who scored 5 or higher.

### 2.5 Safety Assessment

All adverse events were collected and documented up to 12 months posttreatment.

### 2.6 Statistical Analysis

Statistical analyses were conducted using SPSS for Windows version 27.0 (IBM Corp., Armonk, New York). Descriptive statistics, including means for continuous variables and frequency and percentage for categorical variables, are provided throughout. Paired *t*‐tests were used to compare the total facial convexity angle, radix height, and FACE‐Q scores. *p* values were calculated based on two‐sided tests with a significance level set at 0.05.

### 2.7 Tissue Biopsy and Morphological Observation

One patient required additional surgeries near the eyebrow 12 months after the treatment, and the biopsies were taken from the injection site and the adjacent regions. The harvested tissue samples were fixed in 10% neutral‐buffered formalin, followed by paraffin embedding. The embedded tissues were then vertically sectioned along the longitudinal axis with a microtome, creating uniform slices with a thickness of 4 μm. Subsequently, the sections were stained utilizing Hematoxylin and Eosin, Masson's Trichrome, and Modified Russell‐Movat Pentachrome. Visualization was conducted using a light microscope (Model BX63, Olympus Corporation, Tokyo, Japan).

## 3 Result

### 3.1 Participants Disposition and Characteristics

All 15 participants received injections in the eyebrow area, 14 received injections in the nose, and eight received injections in the forehead. The total average injection volume was 3.06 mL (±0.86) overall, with specific averages of 1.42 mL (±0.34) for the eyebrow, 0.77 mL (±0.15) for the nose, and 1.75 mL (±0.89) for the forehead, as detailed in Table 2.

**TABLE 2:** Demographic and Baseline Characteristic.

Variable	
Gender, *n*	
Female	13
Male	2
Age, average ± SD	32.96 (±2.45)
Injection Region, *n*	
Eyebrow	15
Nose	14
Forehead	8
Injection Dose, Average ± SD	
Eyebrow	1.42 (±0.34)
Nose	0.77 (±0.15)
Forehead	1.75 (±0.89)
Total	3.06 (±0.86)

Abbreviation: SD, standard deviation.

### 3.2 Effectiveness

#### 3.2.1 Total Facial Convexity Angle and Radix Height

**FIGURE 2:**
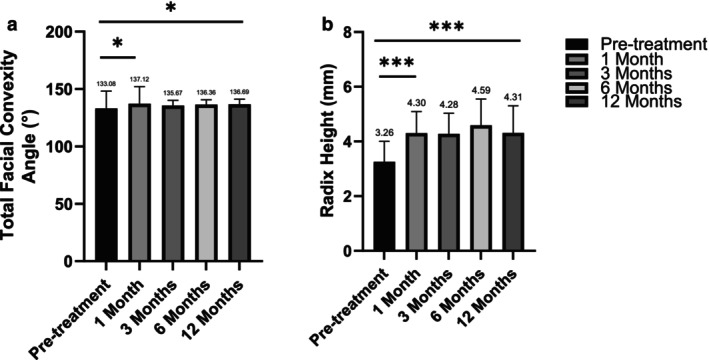
Changes in the total facial convexity angle (a) and radix height (b) at different time points following treatment. (a) The total facial convexity angle shows a significant increase at 1 month posttreatment compared with pretreatment values (*p < 0.05). The values remain stable at 3, 6, and 12 months. (b) The radix height significantly increases at 1 month posttreatment compared to pretreatment levels (***p < 0.001), with values maintained at 3, 6, and 12 months. Data are expressed as mean ± standard deviation (SD). *p < 0.05; ***p < 0.001.

For total facial convexity angle, a significant immediate increase was observed, with the pretreatment average of 133.08° rising to 137.12° at 1 month. Although there was a slight decrease at 3 months to 135.67°, the angle remained consistently above the baseline, showing 136.36° at 6 months and 136.69° at 12 months. Statistical analyses revealed significant differences (*p* < 0.05) between the pretreatment and 1‐month measurements, as well as between the 3‐month, 6‐month and 12‐month post‐measurement (Figure 2a).

In terms of radix height, the pretreatment measurement showed a mean radix height of 3.26 mm, which was increased to 4.30, 4.28, 4.59, and 4.31 mm at 1, 3, 6, 12 months after treatment, respectively. Each time point posttreatment showed a statistically significant improvement (*p* < 0.01) compared with the baseline values (Figure 2b).

**FIGURE 3:**
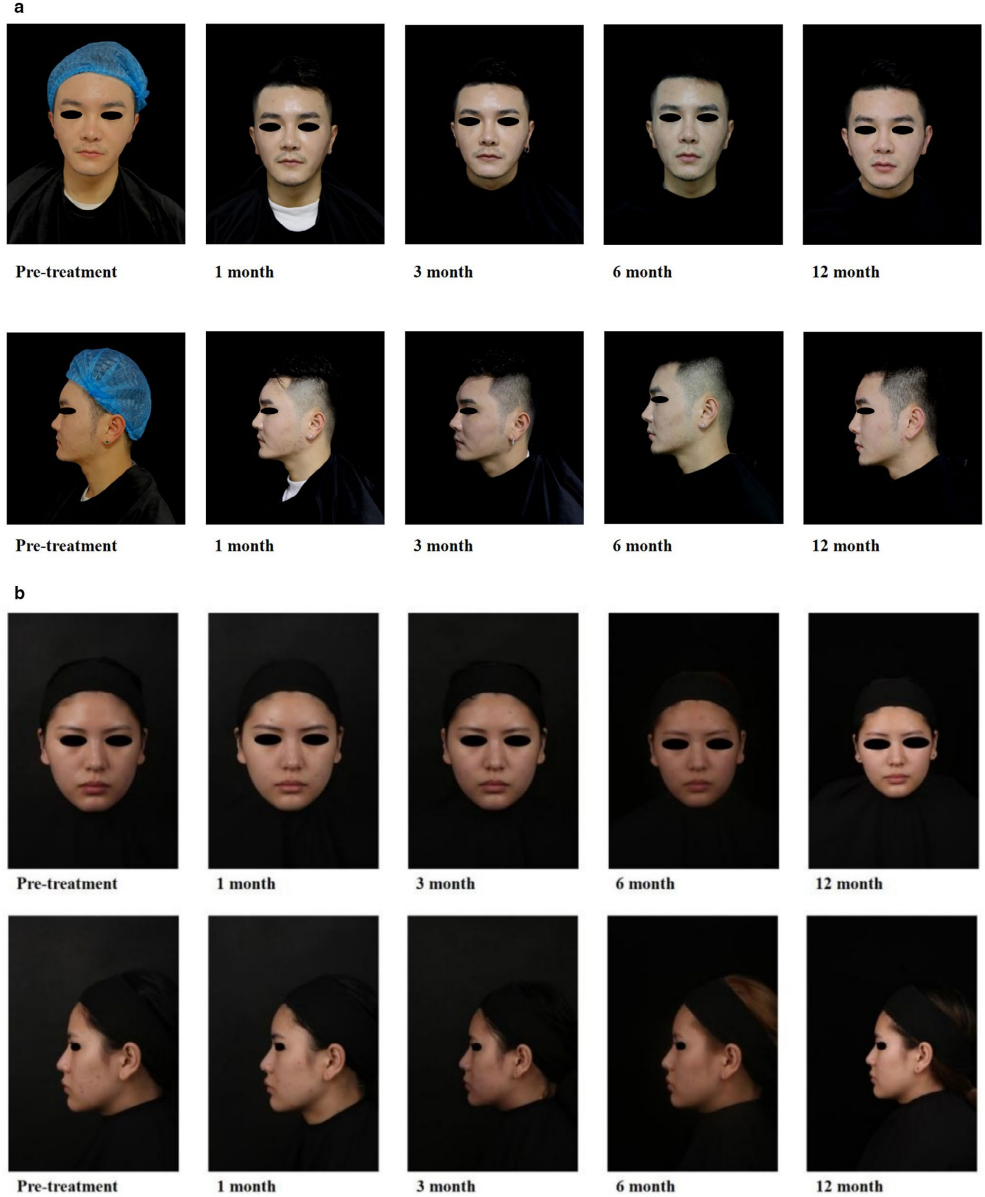
Effects of treatment with cross‐linked sodium hyaluronate gel containing poly‐L‐lactic acid‐b‐PEG microspheres over time. The figure illustrates changes in facial appearance in (a) a 32‐year‐old male and (b) a 30‐year‐old female at different time points: pretreatment, 1, 3, 6, and 12 months posttreatment. The images include both frontal and lateral views, showing improvements in facial convexity and radix height over the observation period.

No significant differences were observed in the total facial convexity angle or radix height across the posttreatment follow‐up visits (all *p* > 0.05). These findings suggest that the treatment effects remained stable throughout the 12‐month follow‐up period, with no notable changes or fluctuations over time (Figure 3).

#### 3.2.2 Face‐Q Scale

**FIGURE 4:**
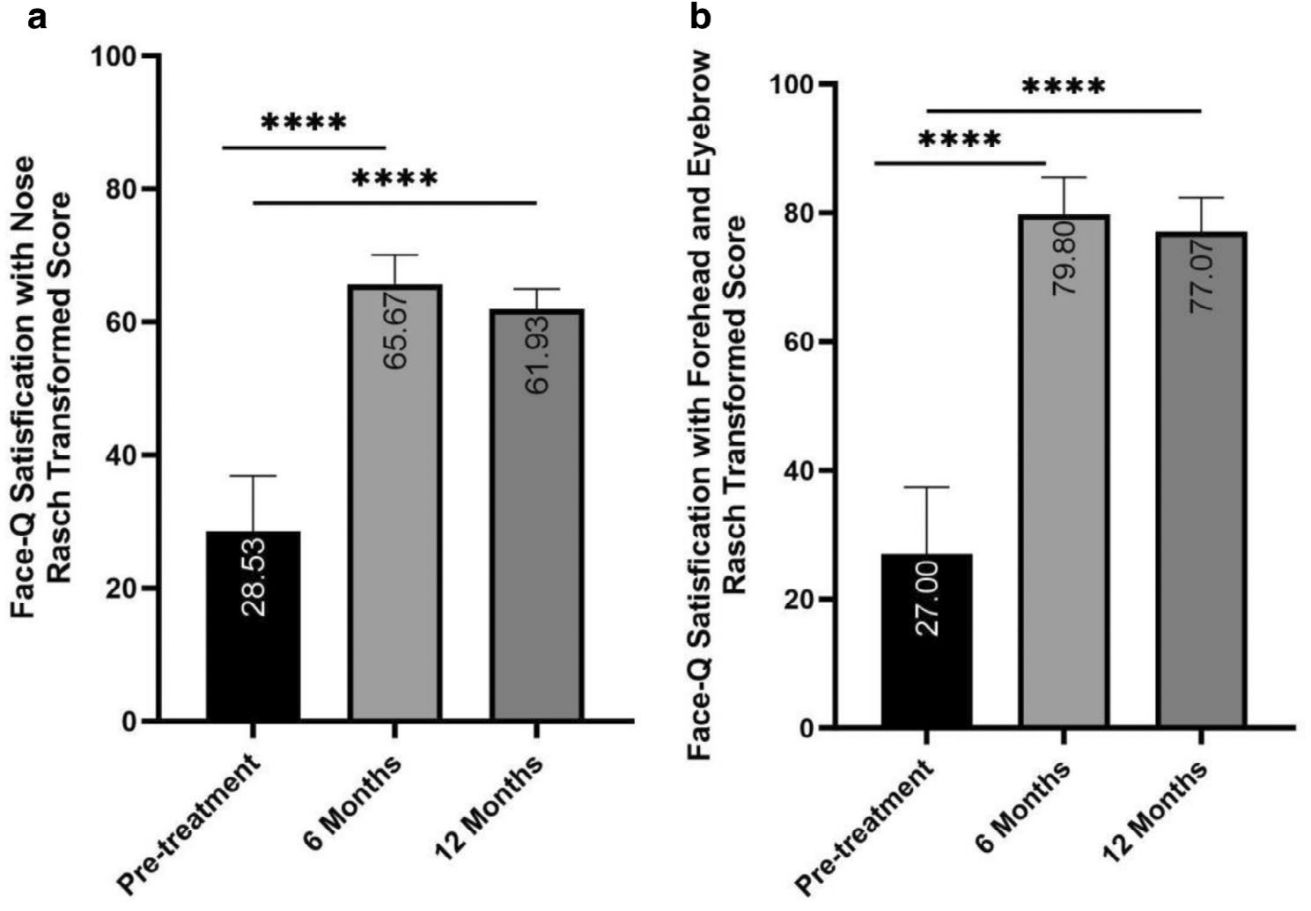
The Rasch‐transformed FACE‐Q satisfaction scores for (a) nose and (b) forehead and eyebrow at pretreatment, 6 months, and 12 months posttreatment. (a) Satisfaction with the nose significantly improved from pretreatment (28.53) to 6 months (65.67) and 12 months (61.93) posttreatment (****p < 0.001). (b) Satisfaction with the forehead and eyebrow also significantly increased from pretreatment (27.00) to 6 months (79.80) and 12 months (77.07) posttreatment (****p < 0.001). Data are presented as mean values with standard error.

The score of FACE‐Q satisfaction with nose was 28.53 (±8.33) at baseline and increased to 65.67 (±4.40) at 6 months posttreatment and decreased slightly to 61.93 (±3.03) at the 12‐month follow‐up (*p* < 0.001) (Figure 4a). Similar trends were also observed in the eyebrow and forehead area. The FACE‐Q satisfaction with eyebrow and forehead mean score was 27.00 (±10.39) at baseline and was improved significantly to 79.80 (±5.70) and 77.07 (±5.34) at 6 and 12 months after injection, respectively (*p* < 0.001) (Figure 4b).

#### 3.2.3 GAIS Improvement Rate

The EI reported a GAIS improvement rate of 93.33% at 1 and 3 months posttreatment, stabilizing at 86.67% at 6 and 12 months after injection. Participants' self‐assessed GAIS improvement rates were 93.33% at 1 and 3 months posttreatment, which decreased to 86.67% at Month 6 and further to 80.00% at Month 12.

**FIGURE 5:**
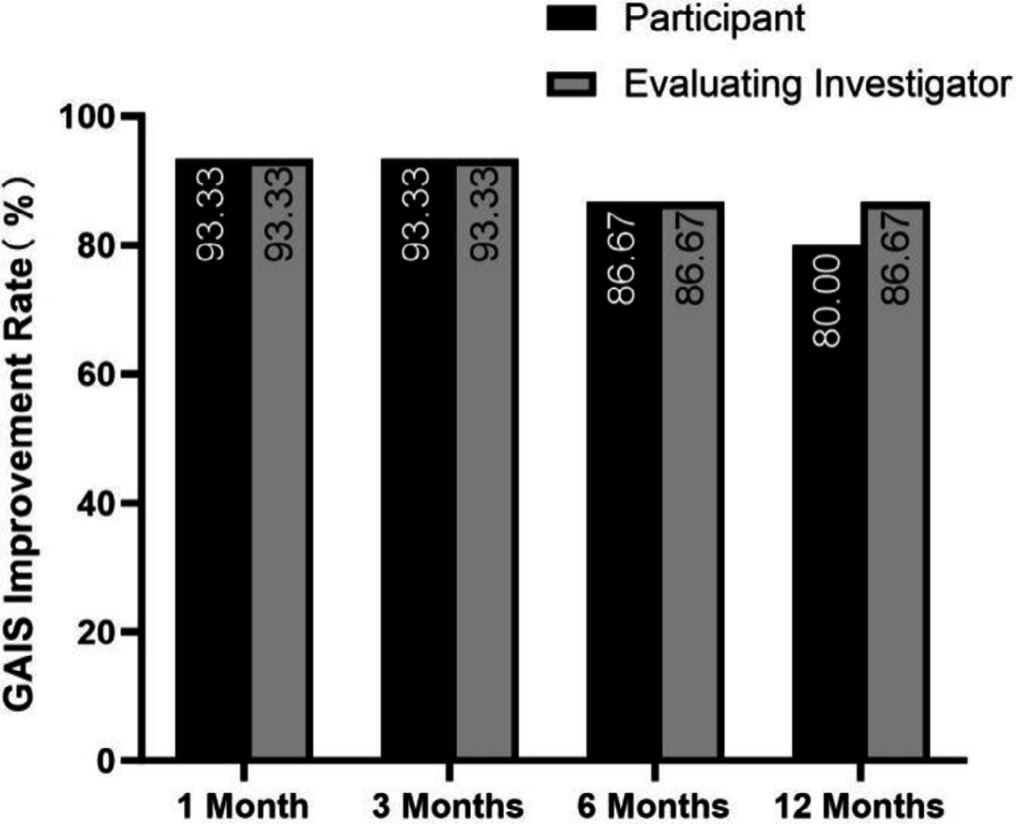
Proportion of participants achieving aesthetic improvement, as assessed by participants (black bars) and the evaluating investigator (gray bars) using the Global Aesthetic Improvement Scale (GAIS) at 1, 3, 6, and 12 months posttreatment. The improvement rate remained high (93.33%) at 1 and 3 months for both participants and investigators. At 6 months, the improvement rate decreased slightly to 86.67% for both groups. By 12 months, participants reported an improvement rate of 80.00%, while the evaluating investigator reported 86.67%.

A slight discrepancy between the EI's and participants' GAIS improvement rates was observed at 12 months posttreatment (Figure 5).

#### 3.2.4 Satisfaction Rate

The treatment achieved a high patient satisfaction rate of 93.33% at the one‐month follow‐up, which gradually declined to 86.67%, 80.00%, and 73.33% at the 3, 6, and 12‐month follow‐ups, respectively. Nonetheless, at no point during the follow‐up visits did any patient report dissatisfaction, with scores ranging from 1 to 3.

### 3.3 Safety

No serious adverse events (SAEs) were reported in this study. Only two participants (13.33%) experienced transient mild redness and swelling at the injection site, both of which resolved without any interventions. Furthermore, no PLLA‐related adverse events, including granulomas, papules, or nodules, were observed.

### 3.4 Long‐Term Histopathological Response After Implantation

**FIGURE 6:**
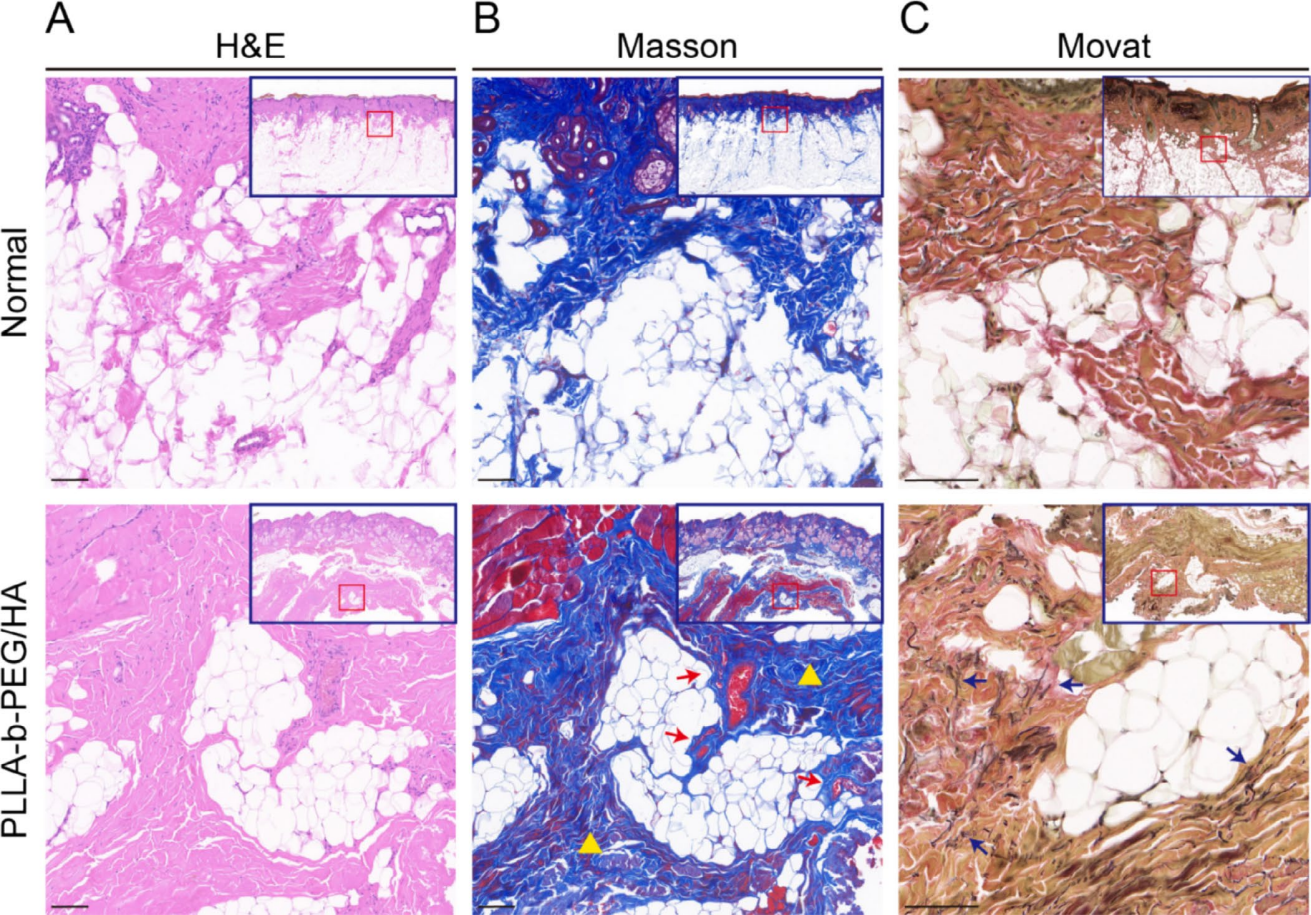
Histological responses were evaluated using hematoxylin and eosin (H&E), Masson's trichrome (Masson), and modified Russell‐Movat pentachrome (Movat) staining. (A) The skin structure in normal tissue exhibited no pathological changes. In the tissue examined 12 months after the implantation of PLLA‐b‐PEG/HA filler, the implant material was no longer present, with no significant inflammatory cell infiltration, although scattered fibroblasts were observed (scale bar: 100 μm). (B) Compare to normal tissue, where the content and arrangement of skin collagen fibers were orderly, the tissue treated with PLLA‐b‐PEG/HA filler injection showed numerous new collagen fibers (yellow triangle), along with newly formed microvessels (red arrow). The collagen arrangement in this area appeared denser (scale bar: 100 μm). (C) Elastic fibers were relatively sparse in normal tissue. Following the injection of PLLA‐b‐PEG/HA filler, a dense network of elastic fibers (blue arrow) was observed within the newly formed collagen tissue (scale bar: 100 μm).

In the normal tissue, skin fibers exhibited an orderly arrangement with no evident pathological alterations. Twelve months after implanting the PLLA‐b‐PEG/HA filler, histological evaluation revealed no presence of residual implant material or significant inflammatory cell infiltration in the treated regions. However, numerous dispersed fibroblasts was observed. Masson staining highlighted a significant presence of newly formed, orderly arranged, blue‐colored collagen fibers within the implant area, which were well‐integrated with the surrounding adipocytes. Additionally, a notable number of newly formed microvessels were observed within these collagen fibers. Movat staining further demonstrated a dense network of elastic fibers within the newly formed collagen tissue. Remarkably, there was an increase in the thickness of the dermal layer above the injection site, accompanied by a denser arrangement of collagen fibers (Figure 6).

## 4 Discussion

The nose, forehead, and eyebrow are components of the “T‐zone” and play pivotal roles in facial aesthetic expression and projection. This is particularly relevant for the Asian demographic, often characterized by a lack of anterior midface projection. Specifically, the forehead often appears less pronounced, the nose is typically smaller and broader with a bulbous, under‐projected tip, the chin appears indistinct, and the mandible is large, sometimes giving a bulky appearance. Enhancements of the T‐zone are therefore critical for augmenting projection and three‐dimensionality [1]. In addition, augmentation of the T‐zone is essential in the gender‐specific emphasis. The differences in ideal facial features between men and women provide a clear framework. For women, nasion injections are particularly important to create a smoother, mildly concave nasal dorsum and a higher nasofrontal angle, which contribute to a softer and more feminine transition between the forehead and nose. Additionally, forehead augmentation should focus on enhancing the natural curvature and reducing any flatness or prominence in the supraorbital area, as the female forehead typically exhibits a continuous and gentle contour. In contrast, for men, injections in the glabellar region may be emphasized to preserve or subtly enhance the prominence of the brow ridge, a defining masculine feature. Nasion augmentation in men should be minimal, as a straighter and more prominent nasal dorsum aligns with the ideal masculine profile. These gender‐specific considerations highlight the importance of injection placement to achieve outcomes that respect the anatomical and esthetic differences between male and female faces.

HA fillers have become the predominant choice for enhancing facial volume and reducing wrinkles, comprising approximately 80% of all fillers used in rejuvenation treatments [19]. Their popularity is largely due to technological improvements that have increased their insolubility, thereby extending their effective duration. Despite these improvements, HA fillers are still naturally degraded and absorbed in the body, and their diffusion and distribution can vary after injection [20]. As an alternative, PLLA is known for its ability to promote the regeneration of fibrous tissues and collagen, maintaining its effects for up to 2 years [21, 22].

The hybrid of PLLA‐b‐PEG and cross‐linked HA is designed to provide both immediate and sustained efficacy. Cross‐linked HA offers instant physical support. As the HA gradually and naturally degrades, the biostimulatory collagen effect of PLLA induces fibroblast proliferation, which secretes collagen to maintain volumetric effects, as supported by the results of this study [23]. Pathological examinations results revealed complete degradation of the PLLA‐b‐PEG/HA filler within the implant site, with significant infiltration of fibroblasts. Furthermore, the newly formed collagen fibers, abundant in newly formed microvessels and elastic fibers, were arranged orderly and exhibited tight integration with the surrounding tissue.

In early clinical applications of PLLA materials for facial soft tissue augmentation, adverse reactions such as nodules and granulomas caused by strong stimulation postimplantation have been a concern for clinicians [24, 25]. Although advancements in material processing and injection techniques have led to a decrease in these adverse reactions, long‐term granuloma formation remains a notable issue [26]. The introduction of PLLA‐b‐PEG/HA filler, formulated through the copolymerization into amphiphilic microspheres, has demonstrated a mild yet persistent tissue response throughout the implantation process, effectively reducing the risk of adverse reactions, as validated by animal studies [22]. Pathological examinations in this study confirmed that 12 months postimplantation, the filler had completely degraded, with newly formed collagen tissue arranged in an orderly manner. These findings underscore the long‐term safety of PLLA‐b‐PEG/HA filler. Furthermore, the microspheres exhibited an induction effect on neovascularization similar to that of traditional PLLA filler [27], suggesting that the new connective tissue could maintain activity over an extended period. Additionally, the opaque, milky‐white composite gel of PLLA‐b‐PEG/HA filler avoids the issue of translucency at the injection sites, thereby ensuring a more natural and esthetically pleasing outcome.

The quality and thickness of the dermis gradually decline with age. Thus, dermal thickening is an important procedure in antiaging treatment. Previous research has established that the PLLA effectively enhances dermal thickness by stimulating the proliferation and regeneration of cutaneous collagen, especially the type I collagen [28]. Consistent with these findings, our study observed that the collagen tissue newly formed by the PLLA‐b‐PEG/HA filler was rich in elastic fibers, and there was significant thickening of the dermal layer above the implantation site. This suggests a potential improvement in the surrounding skin quality.

The efficacy and safety of the PLLA‐b‐PEG/HA filler was proven to be sustained. Participants achieved significant facial convexity and radix height enhancements at the 12‐month mark. While the FACE‐Q satisfaction scores and GAIS ratings remained high at the 12‐month follow‐up. While the EI reported a stable improvement rate of 86.67% at 6 and 12 months, participants' self‐assessed improvement rates declined from 86.67% at 6 months to 80.00% at 12 months. This slight discrepancy between the EI's and participants' assessments might indicate a difference in professional and personal expectations, or it could reflect subtle changes that are more noticeable to the patients themselves. The overall satisfaction rate remained high throughout the study period, with no reports of dissatisfaction. In summary, the PLLA‐b‐PEG/HA filler demonstrates sustained efficacy in the T‐zone augmentation for up to 12 months, with high levels of and long‐term overall facial improvement and safety profile.

This study has several limitations. First, the retrospective design inherently restricts our ability to establish causality and relies on the accuracy of existing records, which may introduce bias or missing data. Second, the small sample size of 15 participants limits the generalizability of the findings and may reduce statistical power. Additionally, the study population may not be representative of broader patient groups, further limiting external validity.

## Author Contributions

Yinxian Qiu, Mengjie Zhu, and Wei Chen performed the research. Shiwei Wang and Muyan Zou designed the study. Wei Chen, Jialun Li, and Yujia Diao contributed essential reagents or tools. Jiaxu Wu, Guanqun Qiao, and Shiwei Wang analyzed the data. Muyan Zou, Jiaxu Wu, and Wei Cai wrote the paper. Each author have participated sufficiently in the work to take public responsibility for appropriate portions of the content; and agreed to be accountable for all aspects of the work. All authors have read and approved the final manuscript.

## Ethics Statement

The study was approved by the Ethics Committee and complied with the ethical requirements of the Declaration of Helsinki.

## Conflicts of Interest

The authors declare no conflicts of interest.

## Data Availability

The data that support the findings of this study are available from the corresponding author upon reasonable request.
